# Functionality of symptoms and interpersonal communication in home video recordings of functional/dissociative versus epileptic seizures

**DOI:** 10.1002/epi.70107

**Published:** 2026-02-05

**Authors:** Nayrin Dissouky, Klara Kochs, Robert Daniel Nass, Tobias Baumgartner, Attila Rácz, Rainer Surges, Markus Reuber, Juri‐Alexander Witt, Christoph Helmstaedter

**Affiliations:** ^1^ Department of Epileptology University Hospital Bonn Bonn Germany; ^2^ Academic Neurology Unit, University of Sheffield, Royal Hallamshire Hospital Sheffield UK

**Keywords:** communicative function, dissociation, epileptic seizures, functional dissociative seizures, home videos

## Abstract

**Objective:**

Conceptualizing functional/dissociative seizures (FDS) as resulting from dissociation, or conversion, we hypothesized that, compared to epileptic seizures (ES), FDS should carry more symbolic or communicative content and that this would allow observers to distinguish FDS from ES.

**Methods:**

Three independent, epileptologically naive raters evaluated home videos of patients with confirmed diagnoses of either FDS or ES using a standardized form. The focus of the ratings was explicitly not on seizure semiology, but on verbal and nonverbal behavior, the role of proxies, interaction patterns, communication, emotional tone, symbolic content, and situational context.

**Results:**

Of 598 home videos available from 183 patients, 215 ES and 95 FDS videos were suitable for analysis. No explicit symbolic communication was identified. FDS showed more passive, withdrawn behavior, and the postictal phase—captured more often than the ictal period—was particularly helpful for distinguishing FDS from ES. Interrater reliability was moderate. Features observed more commonly in FDS included closed eyes, recumbent posture, repetitive movements, reduced eye contact, responses to caring behavior, and occurrence in private settings. Raters perceived greater emotional distress in FDS and reported more distress watching these videos. Logistic regression based on all ratings correctly classified 94% of ES but only 32% of FDS.

**Significance:**

Home video analysis captures important contextual and behavioral features of FDS and ES. The differential diagnostic reliability of lay raters' perceptions is limited. Findings suggest that FDS comprise passive rather than active appellative communication, likely reflecting emotional regulation processes. In contrast, in the home videos studied, ES patients exhibit greater postictal awareness and interaction than FDS patients, pointing to the relevance of the postictal phase for discriminating both seizure types. The results emphasize integrating environmental context and patient–caregiver interactions before, during, and after seizures to understand the functional significance of FDS in naturalistic, nonclinical settings.

**Key points**

- In the examined home videos, FDS patients showed features like eye closure, leg movements, repetitive motions, recumbent posture, and withdrawal.
- ES patients remained upright, alert, and socially engaged after seizures.
- These postictal behaviors especially discriminated between FDS and ES.
- In FDS, caring behaviors sometimes reduced and sometimes reinforced seizures and elicited stronger observer responses.
- FDS displayed dissociative, passive, and distinctive communicative patterns with almost no symbolic communicative features.

## 1 INTRODUCTION

Functional/dissociative seizures (FDS) are characterized by paroxysmal manifestations of abnormal motor, sensory, and mental functioning resembling epileptic seizures (ES), but unrelated to epileptiform electroencephalographic (EEG) ictal changes.^1^, ^2^ FDS are diagnosed in 15%–30% of patients referred to epilepsy centers.^3^ However, the mean latency between onset of FDS and correct diagnosis is several years, with >80% of patients initially receiving inappropriate and potentially harmful antiseizure medication and no effective treatment.^3^, ^4^

The etiology of FDS disorders, previously known as psychogenic nonepileptic seizures (PNES), involves a number of interacting biopsychosocial factors, but the seizures themselves are considered a predominantly psychological reaction to internal or external triggers rather than the results of physiological neurological dysfunction.^5^ However, although psychological stressors may play a role as predisposing or maintaining factors, they are not always identifiable,^6^ and their identification is not required in the most recent diagnostic criteria as listed in the Diagnostic and Statistical Manual of Mental Disorders, 5th edition.^7^ Rather than relying on the exclusion of epilepsy or presence of psychological stressors, increasing emphasis has recently been placed on making the diagnosis of FDS on the basis of positive clinical features such as a typical semiology and on observable signs^8^ as well as communication styles via linguistic analysis.^9^, ^10^ Although descriptions of semiological features may be sought from patients or witnesses, the recording of observable features (e.g., with home video) can make a more reliable contribution to the diagnostic process. However, the gold standard for diagnosing FDS involves the recording of typical seizures with video‐EEG (vEEG)^11^ to exclude an epileptic etiology.

As a consequence of the “standard” diagnostic process, established since the 1980s, most of the criteria that have been described as potentially diagnostic were noted in the context of vEEG recordings in clinical settings.^12^ In contrast, subjective symptoms or the situational and social context in which the seizures typically occur have received less attention from researchers. Only a small number have explored the situational context of FDS. For instance, Wardrope et al.^13^ previously examined FDS in a somewhat homelike, although still clinical environment and demonstrated that peri‐ictal responsiveness to the social environment is greater in FDS than in ES. The presence of others was deemed to have influenced the intensity of 51% of FDS but none of the ES. Other studies comparing FDS and ES have revealed potentially relevant social and emotional differences between these two seizure disorders, providing additional support to the idea that FDS may have a social or communicative function. In this study, we focus on two types of communicative behaviors: “symbolic communication” and “passive appellative communication”. Symbolic communication refers to behavior triggering associations and conveying meaning beyond the simple motor action, whereas passive appellative communication refers to behaviors aimed at attracting attention or eliciting responses from others without active initiation.

FDS vocalizations tend to be complex and emotionally expressive, whereas vocalizations associated with most ES are typically more primitive and lack emotional content,^11^ although seizures involving prefrontal regions can also present with complex or affectively charged vocalizations.^14^ This communicative role is also evident in diagnostic interactions between patients and clinicians; patients with ES focused more on seizure symptoms, whereas those with FDS were often more concerned with their social context or consequences.^15^, ^16^

Traditional ideas that “conversion symptoms” reflect unmet emotional psychological needs, as first formulated by Freud and Breuer in the 19th century and summarized by Chodoff,^17^ still reverberate in more recent interpretations. For example, Martino et al.^18^ proposed that FDS may express emotional content that patients are otherwise unable to communicate, and Beghi et al.^19^ argued for a more intuitive approach to understanding the visible manifestations of FDS. Although the Integrative Cognitive Model of FDS proposed by Brown and Reuber^5^ does not assign an explicit communication function to these seizures, it does interpret these seizures as an automatic expression of distress and arousal. This understanding aligns with the defense cascade model, which, according to Kozlowska et al.,^20^ can manifest in five automatic defense states triggered by high arousal, ranging from more active to more passive reactions: arousal, fight or flight, freeze, tonic immobility, and collapsed immobility.

Although our study focuses on psychological and behavioral features of FDS, emerging evidence highlights measurable nervous system dysfunction, consistent with a biopsychosocial framework. Resting‐state functional connectivity studies show altered network dynamics in FDS/PNES, including disrupted interactions across frontoparietal, sensorimotor, and insular regions.^21^, ^22^, ^23^ Network‐level analyses further demonstrate reduced stability and altered integration across global brain networks during event‐free periods, indicating a baseline vulnerability.^24^ Beyond resting‐state alterations, ictal recordings indicate that FDS events are associated with transient neural network changes.^25^, ^26^ Together, these findings suggest that FDS involve both baseline and dynamic ictal network alterations, complementing the psychological and behavioral perspectives emphasized in our study.

Based on these previous studies and interpretations, we hypothesized that, if FDS represent a conversion symptom, they may convey symbolic or communicative content, which in real‐life settings could be observed as emotional signaling or interpersonal responsiveness. We therefore expected FDS to involve more emotionally expressive behaviors and to generate greater emotional responses in observers than ES, reflecting these observable patterns of communication. To demonstrate communicative potential, such behaviors and responses should be detectable with higher interrater reliability (IRR) in FDS than ES. Table 1 summarizes key findings from previous research.

**TABLE 1:** Key findings in the literature.

	Authors	Findings
Interaction with others	LaFrance et al.^11^	FDS vocalizations tend to be complex and emotionally expressive, whereas vocalizations associated with most ES are typically more primitive and lack emotional content
	Plug et al.^15^, ^16^	Patients with ES are more focused on the seizure itself, whereas those with functional seizures tend to focus less on the seizure and more on its social context or consequences
	Wardrope et al.^13^	The intensity of 51% of FDS (but none of the ES) was judged to be influenced by the presence of others FDS and ES differ in peri‐ictal social responsiveness, especially in interactive settings The presence of others significantly affected individuals with FDS before, during, and after the seizures, compared to ES In natural social settings, others' influence on seizure intensity is a specific clinical marker
Functional role of FDS	Martino et al.,^18^ Chodoff,^17^ Beghi et al.^19^	FDS serve a communicative function, expressing emotional content FDS differ from ES in functionality and the emotional content communicated
	Brown & Reuber^5^	Suggests that the central feature of all FDS is the automatic activation of a mental representation of seizures (the “seizure scaffold”) in the context of a high‐level inhibitory processing dysfunction FDS often arise from elevated autonomic arousal and may disrupt awareness of distressing material, but can become divorced from ongoing autonomic and emotional activity

Abbreviations: ES, epileptic seizures; FDS, functional/dissociative seizures.

To focus on the emotional valence and expressiveness of seizure manifestations in real‐life settings, in this study, we compared ratings of home video recordings of FDS and ES by individuals with no epileptological expertise.

Seizure semiology was not addressed explicitly, because we anticipated that nonexpert raters would be more consistent in the appreciation of the overall nature of a seizure than in recognizing specific semiological features, which typically require extensive training to detect accurately. The impact of caregiver interactions was also explored, with the expectation that FDS patients would be more sensitive to their social environment.^13^

## 2 MATERIALS AND METHODS

A total of 598 videos from 183 patients attending the Department of Epileptology at the University Hospital Bonn, Germany, collected between 2008 and October 2018, were initially reviewed. Patients and their caregivers had been encouraged by clinicians to provide video recordings of their seizures for diagnostic purposes, without specific recording instructions. All patients had consented to their videos being stored and analyzed. Videos were analyzed using the video coding scheme described below. Three psychology master students, confirmed as nonexperts and blinded to both the diagnosis and the study objective, rated all seizure episodes separately in a randomized order.

Naive raters were deliberately chosen to investigate whether functional seizures carry symbolic content or serve specific functions, such as trauma processing. Using nonexperts ensured their judgments were not influenced by prior theoretical or clinical knowledge, reducing bias and capturing intuitive perceptions of seizure behavior. This also minimized potential learning effects or unconscious tendencies, such as associating patient characteristics (e.g., female gender) with FDS. Raters were compensated and encouraged to watch each video fully, repeating viewings as needed for accurate ratings.

### 2.1 Video screening procedure and inclusion criteria

The quality and duration of the videos varied, as they were recorded by seizure witnesses with personal equipment, often missing the seizure onset while setting up the camera. Videos were screened using a modified version of the quality scale suggested by Dash et al.,^27^ with those meeting at least four of 10 criteria included, instead of the original five. The decision to accept videos meeting only four criteria was made because of the study's focus on interactional rather than subtle semiological features. Videos longer than 5 min were shortened by one of the raters, who completed the initial screening of all videos. The modified scale and assessment flowchart are included in Appendix S1.

**FIGURE 1:**
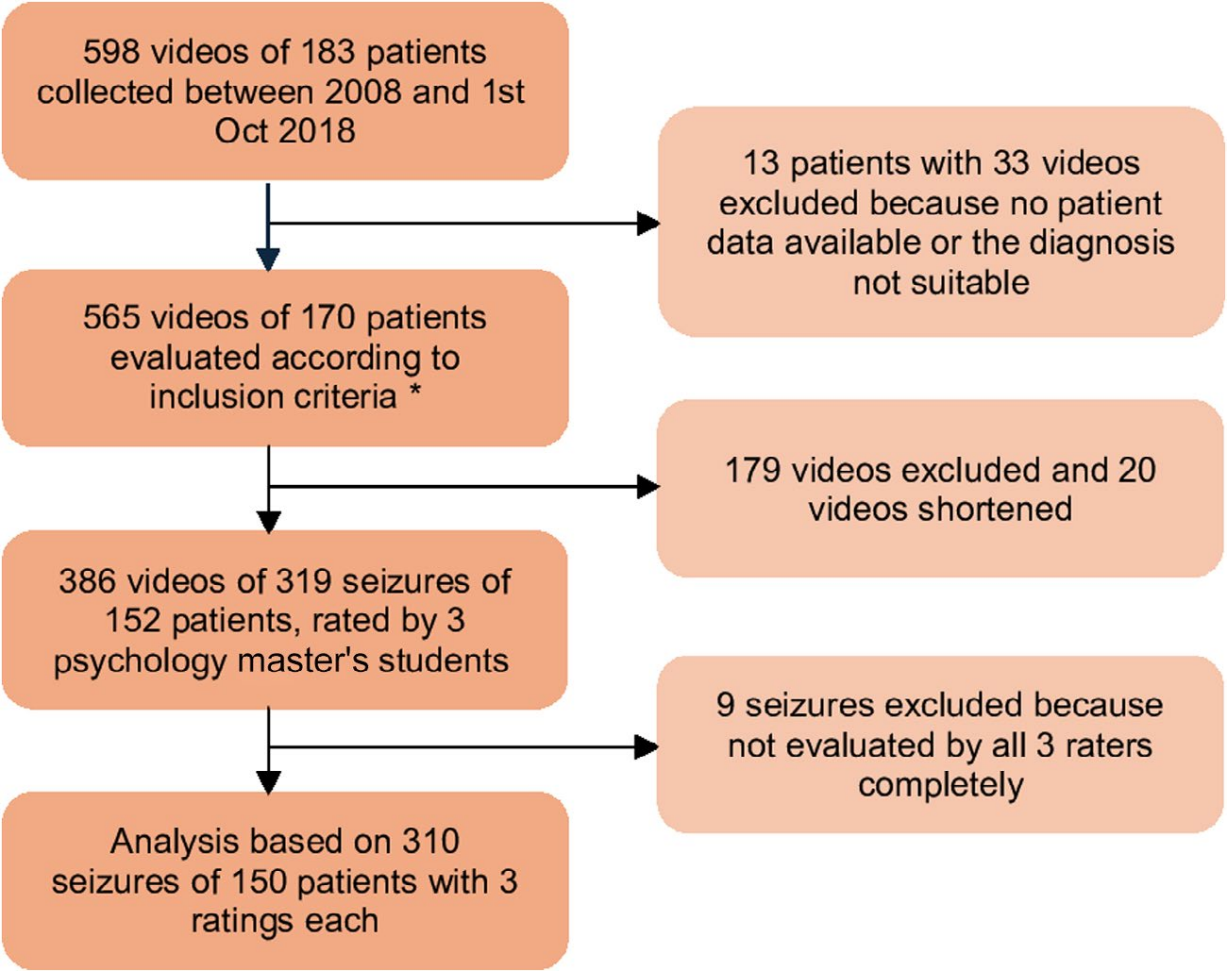
Flowchart of patient and video screening and coding. *See inclusion criteria and screening procedure in the text.

Videos of patients diagnosed with ES, FDS, or both were included, with diagnoses made by trained neurologists based on the videos and all available clinical data (Figure 1). In 75% of cases, the diagnosis was confirmed by vEEG, and in the remaining 25%, it was based on clinical history, EEG, imaging, comorbidities, seizure provocation, family history, medication response, and home video recordings. For patients with unclear diagnoses or both conditions, videos were reviewed and independently categorized by three experienced epileptologists. Videos were excluded if they could not be categorized as showing either ES or FDS or were shorter than 5 s. There was no limit on the number of seizures per patient, so multiple seizures from the same patient were included if available. Sometimes, a single seizure was recorded over several video segments. These segments were later grouped based on the recording dates and analyzed as a whole. In practice, only one patient contributed two separate seizures, whereas all other patients contributed a single seizure each. Therefore, the issue of nonindependent observations is negligible and unlikely to affect the results, and no formal statistical adjustment was deemed necessary.

### 2.2 Video coding scheme

A questionnaire was created for the study, including open, semiopen, and closed questions, collecting both quantitative and qualitative data. Deductive questions were based on existing FDS research, whereas inductive questions were developed from video screenings and informed by observations from daily clinical practice. The questionnaire covered the seizure context, the patient, others present, and raters' responses. The full questionnaire (in German) can be found in Appendix S1.

### 2.3 Quantitative analysis

#### 2.3.1 Information content

IRR was computed for all seizures and again separately for ES and FDS groups to control for diagnosis‐related differences in rater agreement.

We calculated Cohen kappa for each coder pair and averaged the resulting three estimates to obtain an overall index of agreement.^28^, ^29^, ^30^ Each nominal variable was recoded as a dichotomous variable, and Cohen kappa was calculated separately for each option. For nominal variables with a single choice option, one kappa value was calculated, because these categories were mutually exclusive. Kappa values were classified into substantial (*k* ≥ .61), moderate (*k* ≥ .41), and low (*k* < .41) values.^31^

The interrater agreement for ordinal variables was calculated using the intraclass correlation coefficient, with estimates and 95% confidence intervals based on a mean‐rating (*k* = 3), consistency‐agreement, two‐way random‐effects model.^32^, ^33^, ^34^ Values less than .5 were considered weak, values equal to .5 or greater were considered moderate, and values equal to .75 or higher were considered good.^32^

#### 2.3.2 Group differences between ES and FDS

Group differences between ES and FDS were examined for features with at least moderate interrater agreement. As the main interest was on the information content derived from home videos and sufficient IRR was established, all seizure ratings were included as single cases in the analysis, resulting in 930 cases from 310 seizures, which violated the assumption of independence. Cross tabulations and χ^2^ values were calculated for nominal variables, and Mann–Whitney *U*‐tests were used for ordinal variables. Finally, we conducted a binary logistic regression analysis to determine which variables best differentiated between ES and FDS. All variables that appeared to differentiate significantly between ES and FDS were included (see Table 4). A backward method was applied.

## 3 RESULTS

A total of 598 videos of 183 patients were identified as potentially suitable for inclusion. After application of selection criteria and categorization, 215 ES and 95 FDS of 150 patients were analyzed (see Table 2). One hundred two of these patients were diagnosed with ES, 42 with FDS, and six with comorbid ES and FDS (see Table 2). Among the 102 patients with ES, the majority of seizures were focal (90, 88.2%), with the remaining being bilateral tonic–clonic seizures (9, 8.9%). The seizure type was unclear or not reported in three patients (2.9%). Within the category of focal seizures, temporal lobe seizures were most common (66, 73.3%), followed by frontal lobe seizures (11, 12.2%) and other focal localizations (6, 6.7%); the localization was unclear or unreported in seven cases (7.8%). There was no significant difference in age between the patient groups (one‐way analysis of variance [ANOVA], *p* = .552), with ages ranging from 4 to 74 years. There was a higher proportion of women in the FDS group (69%) than in the ES group (44.1%; *p* < .001), resulting in proportionally more seizures from female participants in the FDS group (Pearson χ^2^ test, *p* = .001). The mean number of seizures and videos per patient did not differ significantly between groups (one‐way ANOVA, *p* = .130, *p* = .199). The number of seizures recorded per participant ranged between 1 and 9, whereas the number of videos per participant ranged between 1 and 19. The maximum number of videos exceeded the maximum number of seizures because, in some cases, multiple videos were captured for a single seizure event. Similarly, the duration of videos did not differ between groups (one‐way ANOVA, *p* = .070).

**TABLE 2:** Descriptive data of patient demographics and videos.

	Epilepsy group, *n* = 102	FDS group, *n* = 42	Comorbid, *n* = 6	Difference
Female	44.1%	69.0%	83.3%	*p* = .000
Mean age, years (SD)	36.5 (15.1)	37.2 (13.4)	30.3 (9)	*p* = .552
Seizure recordings	215	95	—	*p = .130*
Seizure recordings of females	46.0%	67.4%	—	*p* = .001
vEEG‐confirmed diagnoses (%)	72.5	66.7	50.0	*p* = .043
Duration of videos, mean (SD)	2′:11″ (3′:02″)	2′:51″ (2′:49″)	—	*p* = .070
Number seizures per person, mean (SD)	2.1 (1.5)	2 (1.3)	3.3 (3.1)	*p* = .130
Number videos per person, mean (SD)	2.5 (2.4)	2.4 (1.8)	4.2 (3)	*p* = .199

*Note*: There are no comorbid diagnoses at the seizure level, because all included seizures were categorized as either ES or FDS.

Abbreviations: FDS, functional/dissociative seizures; vEEG, video‐electroencephalography.

### 3.1 Reliability of video assessments

There were no significant differences in terms of the IRR of judgments made about ES or FDS videos (see Table 3). All ratings of the 10 variables related to the context of the seizures showed at least moderate IRR, with a mean $\bar{k}$ = .63. Among the patient variables, 12 of 31 ratings achieved at least moderate IRR (mean $\bar{k}$ = .32). Five of the nine variables related to other persons present had at least moderate IRR (mean $\bar{k}$= .4), and seven of the 12 interaction process variables showed at least moderate IRR (mean $\bar{k}$ = .38). Among the rated variables, one of four ratings demonstrated at least moderate IRR (*α* = .47). Ratings focusing on seizure state had a low IRR.

**TABLE 3:** Interrater reliabilities.

Variable	Overall IRR	IRR ES only	IRR FDS only
Context variables			
Patient in recumbent position	*κ* = .74^a^	*κ* = .73^a^	*κ* = .73^a^
Patient lying flat	*κ* = .78^a^	*κ* = .80^a^	*κ* = .70^a^
Private or public setting	*κ* = .86^a^	*κ* = .86^a^	*κ* = .85^a^
Background television	*κ* = .69^a^	*κ* = .74^a^	*κ* = .57^b^
Background music	*κ* = .49^b^	*κ* = .48^b^	*κ* = .48^b^
Background conversation	*κ* = .42^b^	*κ* = .39	*κ* = .47^b^
Day or night	*κ* = .48^b^	*κ* = .47^b^	*κ* = .50^b^
Other persons present	*κ* = .75^a^	*κ* = .72^a^	*κ* = .81^a^
Pets present	*κ* = .70^a^	*κ* = .72^a^	*κ* = .67^a^
Contact between patient and pet	*κ* = .41^b^	*κ* = .43^b^	*κ* = .21
Patient variables			
Legs moving	*κ* = .58^b^	*κ* = .57^b^	*κ* = .62^a^
Repetitive movement	*κ* = .46^b^	*κ* = .49^b^	*κ* = .31
Eyes closed	*κ* = .61^a^	*κ* = .52^b^	*κ* = .65^a^
Patient seems awake	*κ* = .50^b^	*κ* = .53^b^	*κ* = .53^b^
Patient is producing sounds	*κ* = .59^b^	*κ* = .56^b^	*κ* = .66^a^
Patient uses words	*κ* = .68^a^	*κ* = .72^a^	*κ* = .55^b^
Content of vocalization	*κ* = .48^b^	*κ* = .46^b^	*κ* = .52^b^
Emotional outburst	*κ* = .53^b^	*κ* = .49^b^	*κ* = .60^b^
Emotional valence	*α* = .74^b^	*α* = .72^b^	*α* = .76^a^
Emotional arousal	*α* = .69^b^	*α* = .69^b^	*α* = .68^b^
Emotional direction	*α* = .68^b^	*α* = .74^b^	*α* = .39^b^
Variables relating to other persons			
Others producing sounds	*κ* = .72^a^	*κ* = .70^a^	*κ* = .75^a^
Content of vocalization: seizure (if sound yes)	*κ* = .54^b^	*κ* = .53^b^	*κ* = .54^b^
Emotional valence	*α* = .51^b^	*α* = .55^b^	*α* = .47
Emotional arousal	*α* = .52^b^	*α* = .51^b^	*α* = .55^b^
Emotional direction	*α* = .67^b^	*α* = .65^b^	*α* = .69^b^
Interactional variables (only if other person present)			
Other talking to the patient	*κ* = .62^a^	*κ* = .58^b^	*κ* = .71^a^
Other talking about patient	*κ* = .53^b^	*κ* = .53^b^	*κ* = .53^b^
Patient answering or reacting	*κ* = .59^b^	*κ* = .54^b^	*κ* = .69^a^
Nonverbal and verbal interaction	*κ* = .47^b^	*κ* = .47^b^	*κ* = .47^b^
Eye contact	*κ* = .43^b^	*κ* = .43	*κ* = .43^b^
Body contact	*κ* = .64^a^	*κ* = .63^a^	*κ* = .67^a^
Caring behavior	*κ* = .54^b^	*κ* = .48^b^	*κ* = .68^a^
Rater‐related variables			
Emotional valence	*α* = .47^b^	*α* = .46^b^	*α* = .48^b^

*Note*: The different classification of IRR values for Cohen kappa and Cronbach alpha as described in the Materials and Methods section.

Abbreviations: ES, epileptic seizures; FDS, functional/dissociative seizures; IRR, interrater reliability.

^a^ High interrater reliability.

^b^ Moderate interrater reliability.

### 3.2 Univariate analyses of group difference between FDS and ES

As shown in Table 4, ES patients were more often alone (Variable 10; 3.4% in ES, 1.1% in FDS), recording themselves. Videos showed ES patients more often in an upright position (Variable 1; 47.3% in ES, 29.5% in FDS), whereas FDS patients were more likely to be seen in a recumbent position (Variable 2; 43.1% in ES, 63.9% in FDS). FDS were more frequently recorded in private settings (Variable 3; 81.9% in ES, 88.1% in FDS) with background music (Variable 5; 3.4% in ES, 7.4% in FDS). Pets appeared more often in FDS recordings (Variable 11; 3.1% in ES, 7.7% in FDS). In addition, leg movements (Variable 13; 36.9% in ES, 45.7% in FDS), repetitive motions (Variable 14; 50.4% in ES, 66.3% in FDS), and closed eyes (Variable 15; 26.7% in ES, 60.5% in FDS) were shown more often in FDS recordings. Postictally, ES patients appeared awake (variable 16; 91.3% in ES), spoke more often (Variable 18; 39.8% in ES, 25.3% in FDS), and were more likely to maintain eye contact (Variable 28; 72.9% in ES, 45.3% in FDS).

**TABLE 4:** Univariate analyses for differences between groups.

Variable/question	*n*	% in ES	Level of the individual rating			
			% in FDS	χ^2^	*p*‐value	Cramer *V*
1. Upright position	930	47.3	29.5	25.78	<.001***	.166
2. Recumbent position	930	43.1	63.9	34.08	<.001***	.191
3. Private environment	909	81.9	88.1	4.36	.037*	.069
4. Television in background	930	20.6	22.1	.26	.602	.017
5. Music in background	930	3.4	7.4	7.02	.011*	.087
6. Conversation in background	930	5.7	7.0	.56	.460	.025
7. Daytime	896	85.5	83.0	.91	.363	.032
8. One person present	930	64.3	67.7	1.00	.332	.033
9. >1 person present	930	32.2	31.2	.10	.819	.010
10. Patient alone	930	3.4	1.1	4.20	.047*	.067
11. Pets present	930	3.1	7.7	9.78	.003**	.103
12. Contact between pet and patient	42	45.0	54.5	.38	.758	.095
13. Legs moving	836	36.9	45.7	5.52	.020*	.081
14. Repetitive movement	836	50.4	66.3	17.46	<.001***	.145
15. Eyes closed^a^	862	26.7	60.5	88.50	<.001***	.320
16. Patient seems awake^a^	930	91.3	75.8	40.97	<.001***	.210
17. Patient producing sounds^a^	930	65.4	51.6	15.96	<.001***	.131
18. Patient using words^a^	568	39.8	25.3	6.51	.007**	.107
19. Patient talking about seizures^a^	930	21.1	13.7	7.09	.004**	.087
20. Patient talking about filming	930	5.3	4.9	.05	.874	.007
21. Emotional outburst patient^a^	930	15.3	17.2	.50	.269	.023
22. Others producing sound	905	71.6	68.1	1.15	.306	.036
23. Others talking about seizure	930	38.4	43.5	2.11	.147	.048
24. Others talking to patient	930	55.5	54.0	.17	.721	.014
25. Others talking about patient	930	13.3	17.2	2.37	.130	.051
26. Patient responsive^a^	505	63.1	58.2	1.08	.173	.046
27. Nonverbal & verbal interaction	930	61.9	55.1	3.77	.059	.064
28. Eye contact between patient & others/camera	723	72.9	45.3	48.86	<.001***	.260
29. Body contact between patient & others	897	36.1	42.1	2.94	.088	.057
30. Caring behavior	896	67.8	64.4	1.01	=.320	.033

*Note*: Seizure level is only computed for cases with consistent answers.

Abbreviations: ES, epileptic seizures; FDS, functional/dissociative seizures.

^a^ One‐tailed *t*‐test.

* *p* < .05.

** *p* < .01.

*** *p* < .001.

Raters observed patients expressing greater emotional distress in FDS (Variable 1; *p* = <.001, *r* = −.12) but lower arousal than in ES (Variable 2; *p* = .028, *r* = −.07). The emotional response of raters generated by watching the seizure recordings differed significantly between FDS and ES videos. Watching FDS videos elicited more distress in raters (Variable 7; *p* = .031, *r* = −.07; see Table 5).

**TABLE 5:** Mann–Whitney *U*‐test for group differences in the external assessment of emotions.

	Mean rank, FDS	Mean rank, ES	*U*‐value	*z*‐value	*p*‐value	Effect size, *r*
1. Patient valence	412.87	474.64	76 527.50	−3.51	<.001**	−.12
2. Patient arousal	426.57	466.70	79 508.50	−2.19	.028*	−.07
3. Patient direction	458.48	453.46	86 914,50	−.29	.770	−.00
4. Others valence	461.18	453.02	86 097.50	−.49	.628	−.02
5. Others arousal	455.53	452.61	86 381.00	−.17	.864	−.00
6. Others direction	469.27	448.81	83 249.50	−1.21	.223	−.04
7. Rater valence	430.62	468.02	81 384.00	−2.15	.031*	−.07

*Note*: All *p*‐values are two‐tailed.

Abbreviations: ES, epileptic seizures; FDS, functional/dissociative seizures.

* *p* < .05.

** *p* < .01.

#### 3.2.1 Multivariate analyses

Different from the univariate analysis, where three ratings were used per seizure (930 cases from 310 seizures), the multivariate analysis was based on all valid ratings with complete data. After including these ratings, the dataset comprised 399 seizure videos that were available for binary logistic regression. Table B1 lists the variables included in the final model. Overall, the model correctly predicted 78.9% of the medical expert seizure categorizations, with ES being predicted more accurately than FDS (94% vs. 32%; see Table B2). Notably, 68% of FDS were misclassified as ES. The significant predictors “closed eyes” and “eye contact” were significantly correlated (*r* = −.313, *p* = .000).

## 4 DISCUSSION

The main objective of this study was to explore patient and caregiver behavior and interaction derived from home videos of FDS in comparison to ES, to determine whether FDS exhibit features indicative of dissociation or conversion, and whether they differ from ES in having communicative content with a symbolic, functional, and appellative character.

Despite the to be expected poor‐to‐moderate IRR of deliberately chosen naive raters whose intuitive impressions we wanted to explore, consistent patterns could still be identified. A number of observations made by nonexpert raters blinded to patients' medical diagnoses in the seizure videos (such as closed eyes, leg movements, repetitive motions, and recumbent posture) were found to be associated with FDS. In contrast, observations such as the absence of others in the video and patients being upright, being postictally alert, making eye contact, and speaking turned out to be associated with the medical diagnosis of epilepsy. The observation of predominantly closed eyes in FDS patients aligns with prior findings indicating that a subset of patients with FDS exhibit innate defense responses during seizure episodes,^35^ reinforcing the notion that elements of the defense cascade are a prominent feature of these seizures, although similar defensive behaviors have also been reported in some ES.^14^

Upon regaining awareness, patients with ES were typically reactive and communicative. In contrast, the transition from the ictal to the postictal phase was less clearly defined in patients with FDS, who often appeared dazed and withdrawn. ES patients demonstrated greater social engagement, making more eye contact, speaking more frequently, and consequently receiving more interaction‐related comments from raters. FDS patients, however, displayed more passive communication, with a seemingly reduced capacity to react, leading to diminished communicative exchanges.

Raters perceived FDS patients as more distressed; this was expressed through withdrawal, closed eyes, and reduced responsiveness rather than overt emotional behavior. Importantly, raters also reported feeling more distressed themselves when watching FDS videos compared to ES videos.

Overall, our findings contradict the initial hypothesis that FDS would show more active communicative or appellative features than ES. Instead, FDS patients appeared less open to interaction, potentially reflecting underlying defensive or dissociative states. Although their behaviors lacked explicit symbolism, they required greater interpretive effort from raters, leading to more extensive observational notes. In contrast, ES patients displayed clearer behaviors and full postictal awareness, often speaking readily about their seizures. These findings provide insights that can guide clinicians' diagnostic reasoning and support targeted practitioner training to enhance the accurate assessment and interpretation of seizure behaviors.

Regarding awareness, our findings contrast with Bell et al.,^36^ who reported that FDS patients were more responsive and aware than individuals with focal impaired awareness seizures. This discrepancy likely reflects methodological differences, as their study examined ictal behavior during vEEG monitoring, whereas our data predominantly capture postictal behavior in home recordings. Although FDS patients in our sample appeared less aware—often with closed eyes and reduced responsiveness—previous studies have shown that they may retain richer postevent memory than ES patients.^36^, ^37^ This paradox highlights a dissociation between outward behavior and internal experience^38^, ^39^ and may explain why observers often overestimate impairment.^40^ These findings highlight the need to integrate objective observation with patient‐reported experiences. Ictal testing of awareness, responsiveness, and memory helps differentiate epileptic seizure types.^41^ Similarly, systematic approaches may improve understanding of internal versus external awareness in FDS.

Emotional distress and arousal represented another key dimension of differentiation. Raters not only perceived greater distress in FDS patients during seizures but also reported experiencing more negative emotions themselves after watching FDS videos. Despite this, and contrary to our hypothesis, ratings of intense emotional expression did not differ significantly between groups. The greater reliability of emotional valence ratings for ES videos suggests that the distress in FDS, although potentially greater, is often internalized and not clearly manifest. Furthermore, observers rated arousal levels lower in FDS patients than in ES patients. The finding that the raters' own emotional responses were more insightful than their evaluations of the patients' emotions indicates that FDS nonspecifically elicits empathetic or emotional reactions from observers.

This distinction highlights the diagnostic value of the ictal–postictal transition. In our study, ES patients typically resumed full wakefulness rapidly, whereas FDS patients often remained in a prolonged somnolent state. However, as most ES were focal and 73.3% of patients in the ES group had temporal lobe epilepsy, this finding may reflect the specific seizure types and localization represented in our cohort rather than a general characteristic of ES. Interestingly, content analysis revealed no consistent intergroup differences in seizure‐related conversation, physical contact, or caring behavior, contrary to initial expectations.

Notably, caring behaviors sometimes appeared to reduce seizure manifestations in FDS, whereas at other times they seemed to prolong or intensify them. This variability may reflect different dispositions in FDS: on the one hand expecting supportive or soothing behaviors that downregulate arousal and facilitate recovery from a shutdownlike state, on the other hand entering a defensive mode that pushes others away. Certain forms of engagement may therefore unintentionally provoke defensive withdrawal or immobility, potentially prolonging the episode. These observations align with findings from Wardrope et al.,^13^ who showed that individuals with FDS are more responsive to their social environment than those with ES. Together, these data support the hypothesis that FDS expression may be shaped by an interplay between internal defensive mechanisms and the surrounding social environment, although this cannot be directly confirmed by behavioral observation alone.

### 4.1 Limitations

A major strength of this study is its large, real‐world dataset, including 310 home‐recorded seizures from 150 patients, providing substantial ecological validity. To minimize clinical bias, we employed nonexpert raters, capturing intuitive perceptions of FDS, uninfluenced by prior clinical assumptions. This design allowed us to investigate whether FDS present with distinctive features from a naive perspective.

However, this methodological choice also defined key limitations. As indirect observers, the raters could not fully apprehend the events' intensity, and their reports were necessarily based on impression rather than direct experience. This was reflected in the quantitative outcomes, where moderate IRR was observed for nuanced social cues such as eye contact (*κ* = .43). Rather than merely weakening specific contrasts, this finding is analytically informative; it objectively quantifies the inherent subjectivity in interpreting subtle social communication, a core component of the FDS phenotype we sought to investigate.

The home video methodology presented several challenges. Most recordings missed seizure onset, limiting analysis of potential triggers, and often captured only the postictal phase due to delays in retrieving and activating the recording device. Inconsistent video quality—caused by smartphone limitations, motion blur, or poor lighting—frequently forced raters to infer interactions from audio cues or partial movements. The filmer's emotional state and contextual information varied, caregiver filming skills differed, and recording may have influenced the seizure. Future studies should consider whether caregiver knowledge of the diagnosis affects filming behavior or interactions.

Furthermore, a selection bias favoring ES was present, both in the standard population of a university epilepsy center and in the reliance on voluntarily submitted videos. Furthermore, we acknowledge that certain sample and methodological characteristics may have influenced raters' perceptions. A selection bias favoring ES reflected the typical patient population of a university epilepsy center and may have been reinforced by relying on videos voluntarily submitted by patients. Along with a gender imbalance (more women in the FDS group, consistent with its epidemiology) and variations in video quality, these factors may have subtly shaped judgments of communicative intent. For example, differences in expressive style or reduced visual clarity could introduce bias that, although not quantified here, should be considered when interpreting raters' impressions.

Methodologically, although FDS subtypes (hyperkinetic vs. paucikinetic) were not recorded, as our focus was interactional rather than semiological, future work should address these subtype‐specific differences. Our approach underscores a critical point: relying solely on semiological reports from nonmedical observers—although common in practice—has been repeatedly shown to be unreliable.^42^ This highlights the necessity of supplementing witness accounts with standardized video analysis.

Building on these findings, future studies should employ approaches that incorporate standardized measures, validated questionnaires, and formal rater training to capture more accurate interactional dynamics. The use of implicit assessment tools could provide deeper insights into patient perceptions. A promising avenue for future research is the use of ecological, home‐based, or environmental video recordings to assess FDS. The filming process and the relationship between the filmer and patient may influence seizure expression, as the presence or reactions of familiar others can shape behavioral manifestations.^13^ Capturing contextual variables, such as bystander or caregiver behavior, could provide valuable insights into seizure dynamics and be quantified using automated video analysis and artificial intelligence‐based approaches to support diagnostic prediction.^43^ For future home video research, practical considerations, including seizure onset, stable framing, and adequate lighting, will be important to optimize manual and automated analyses.

## 5 CONCLUSIONS

Despite these methodological considerations, this study identified distinct postictal behavioral profiles. Patients with FDS showed characteristic features such as eye closure, leg movements, repetitive motions, and a recumbent posture. In contrast, patients with ES tended to remain upright, alert, and socially engaged. Furthermore, FDS patients displayed reduced responsiveness, diminished communication, and prolonged withdrawal, behaviors suggestive of defensive or dissociative states.

Interaction‐related behaviors also varied; whereas caring behaviors sometimes reduced seizure expression, at other times they appeared to reinforce it, underscoring the complex interplay between internal psychological mechanisms and the social environment. Moreover, FDS seizures elicited stronger emotional responses from observers.

Although no explicit symbolic communication was identified, the findings highlight key dissociative features, passive behavioral patterns, and distinctive communicative differences between groups, with FDS patients presenting as more withdrawn and less responsive than ES patients. Notably, the postictal phase emerged as particularly discriminative for differentiating between ES and FDS.

## AUTHOR CONTRIBUTIONS

*Conceptualization:* Markus Reuber, Juri‐Alexander Witt, and Christoph Helmstaedter. *Data curation:* Klara Kochs, Robert Daniel Nass, Tobias Baumgartner, Attila Rácz, Juri‐Alexander Witt, and Christoph Helmstaedter. *Patient diagnosis:* Robert Daniel Nass, Tobias Baumgartner, and Attila Rácz. *Data analysis:* Klara Kochs and Christoph Helmstaedter. *Visualization:* Nayrin Dissouky and Klara Kochs. *Writing—original draft:* Nayrin Dissouky, Klara Kochs, Markus Reuber, and Christoph Helmstaedter. *Writing—review & editing:* Nayrin Dissouky, Klara Kochs, Robert Daniel Nass, Tobias Baumgartner, Attila Rácz, Rainer Surges, Markus Reuber, Juri‐Alexander Witt, and Christoph Helmstaedter. *Supervision:* Markus Reuber and Christoph Helmstaedter. All authors have read and agreed to the published version of the manuscript.

## CONFLICT OF INTEREST STATEMENT

The authors declare no potential conflict of interest. We confirm that we have read the Journal's position on issues involved in ethical publication and affirm that this report is consistent with those guidelines.

## Supporting information


Appendix S1.

## Data Availability

The data that support the findings of this study are available from the corresponding author upon reasonable request. The data are not publicly available, as they contain information that could compromise the privacy of research participants.
