# Supplementary material for: Functionality of symptoms and interpersonal communication in home video recordings of functional/dissociative versus epileptic seizures
Source: Epilepsia. 2026 Feb 5;67(5):2241–53. doi: 10.1002/epi.70107 (PMC13179661; doi:10.1002/epi.70107)
Supplement: Supplementary file 1 — Appendix S1. [file EPI-67-2241-s001.zip › epi70107-sup-0001-Appendix.docx]

Appendix A

## The Video Coding Scheme

**Start of Block: Default Question Block**

Anhand der Videos, die Du betrachten wirst, wollen wir den Anfall in seinem natürlichen Kontext untersuchen. Die Videos sind in der Regel von Angehörigen und Vertrauenspersonen der Patienten aufgenommen worden. Sie zeigen den Anfall eines Patienten innerhalb seines privaten Umfelds. Daher können wir Aspekte betrachten, die sich nicht bei Anfällen sehen lassen, die stationär aufgenommen wurden.
Bitte achte nicht nur auf den Patienten. Versuche das gesamte Bild, inklusive anderer Personen und dem Hintergrund, vor dem sich der Anfall ereignet, zu erfassen. Dieses Gesamtbild lässt sich nicht während eines Krankenhausaufenthaltes erfassen, daher ist diese Studie eine wichtige Erweiterung. Bitte nimm auch Deine eigenen Gedanken und Gefühle während des Betrachtens wahr. Wenn Dir Bilder, Vergleiche oder andere Beschreibungen einfallen, nenne diese bitte im dafür vorgesehenen Feld. Schau Dir bitte zunächst das Video vollständig an und beantworte anschließend die Fragen. Manchmal gibt es mehrere Videos zu einem Anfall, dies ist deutlich gekennzeichnet. In diesem Fall schaust du Dir bitte alle Videos zu diesem Anfall an und trägst auch alle Kennziffern in das vorgesehene Feld.

Q59 Gib deine Kennnummer ein:

________________________________________________________________

Q62 Welche(s) Video(s) schaust du dir an? (wenn vohanden mit Kleinbuchstaben angeben, wie z.B. 136a, 136b, 136c)

________________________________________________________________

Q1 In welcher Position befindet sich der Patient?

- aufrecht sitzend oder stehend (1)
- horizontal liegend, halb sitzend (2)
- wechselnd (3)
- nicht sichtbar (4)

Q2 In welcher Umgebung befindet sich der Patient?

- privat, zuhause (1)
- öffentlich (2)
- unklar (3)

Q3 Was passiert im Hintergrund?

- TV (1)
- Musik (2)
- Gespräch (3)
- Anderes, bitte definiere: (4) _______________________________________________
- nichts / kann ich nicht sagen (5)

Q4 Welche Tageszeit ist es? (vermutlich)

- Tagsüber (1)
- Nachts (2)
- kann ich nicht sagen (3)

Q5 Sind neben dem Patienten noch andere Personen anwesend? (hier zählt auch die filmende Person)

- Ja, eine Person (1)
- Ja, mehr als eine - Wie viele? (2) ___________________________________________
- Nein (3)

Q6 Sind Haustiere sichtbar?

- Ja (1)
- Nein (2)

*Display This Question: If Sind Haustiere sichtbar? = Ja*

Q7 Hat das Haustier Kontakt mit dem Patienten?

- Ja (1)
- Nein (2)

*Display This Question: If Sind Haustiere sichtbar? = Ja*

Q8 Beschreibe das Verhalten des Haustieres.

________________________________________________________________

Q9 Bitte beschreibe, wie die Gesamtsituation auf dich wirkt. Du kannst hier alle Bilder, Vergleiche oder Beschreibungen nennen, die bei dir erzeugt werden.

________________________________________________________________

Q10
Liegt der Patient völlig regungslos da, ohne jegliche Bewegung?

- Ja (1)
- Nein (2)

*Display This Question: If Liegt der Patient völlig regungslos da, ohne jegliche Bewegung? = Nein*

Q11 Welche Körperteile bewegen sich?

- Gesichtsmimik (1)
- Kopf (2)
- Rumpf (3)
- Arme (4)
- Beine (5)

*Display This Question: If Liegt der Patient völlig regungslos da, ohne jegliche Bewegung? = Nein*

Q12 Ist die Bewegung auf eine Körperhälfte beschränkt?

- Ja, nur rechts oder links (1)
- Ja, nur oben oder unten (2)
- Nein (3)

*Display This Question: If Liegt der Patient völlig regungslos da, ohne jegliche Bewegung? = Nein*

Q13 Gibt es Bewegungen, die sich wiederholen?

- Ja (1)
- Nein (2)

*Display This Question: If Liegt der Patient völlig regungslos da, ohne jegliche Bewegung? = Nein*

Q14 Verläuft die Bewegung der verschiedenen Körperteile synchron?

- Ja (1)
- Nein (2)

*Display This Question: If Liegt der Patient völlig regungslos da, ohne jegliche Bewegung? = Nein*

Q15 Verändert sich die Intensität der Bewegung?

- Ansteigend (1)
- Abflachend (2)
- kommt und geht in Wellen, dynamisch (3)
- keine Änderung (4)

*Display This Question: If Liegt der Patient völlig regungslos da, ohne jegliche Bewegung? = Nein*

Q16 Bewegt sich der Körper oder Kopf von Seite zu Seite?

- Ja (1)
- Nein (2)

Q17 Sind die Augen des Patienten verschlossen?

- Ja (1)
- Nein (2)
- nicht sichtbar (3)
- zum Teil (4)

Q18 Verletzt der Patient sich offensichtlich während des Anfalls?

- Ja (1)
- Nein (2)

Q19 Gibt es sichtbare körperliche Reaktionen?

- Erröten (1)
- Erblassen (2)
- Schwitzen (3)
- Hyperventilation (4)
- keine (5)

Q20 Ist der Patient wach?

- Ja (1)
- Nein (2)

Q21 Welche Beschreibung(en) passen auf den Patienten?

- Der Patient erscheint der Situation zu entfliehen. (1)
- Der Patient wirkt wütend. Möglicherweise schlägt er um sich und wehrt sich. (2)
- Der Patient ist bewegungslos, aber hat einen angespannten Körper. Er ist bereit für Aktion. Er ist besonders aufmerksam. (3)
- Der Patient ist bewegungslos und erscheint nicht in der Lage sich zu bewegen. Sein Körper ist schlaff. Er wirkt dissoziiert. (4)
- keins davon (5)

Q22
Erzeugt der Patient Geräusche und/oder spricht?

- Ja (1)
- Nein (2)

*Display This Question: If Erzeugt der Patient Geräusche und/oder spricht? = Ja*

Q23 Entnimmst du den Geräuschen emotionale Bedeutung?

- Ja (1)
- Nein (2)

*Display This Question: If Erzeugt der Patient Geräusche und/oder spricht? = Ja*

Q24 Drückt der Patient sich in Worten aus?

- Ja (1)
- Nein (2)

Q26 Schätze im Folgenden die Emotionen des Patienten ein.

Die Valenz: Ist die Wertigkeit der Emotion positiv oder negativ?

- sehr unangenehm (1)
- eher unangenehm (2)
- Neutral (3)
- eher angenehm (4)
- sehr angenehm (5)

Q28 Das Arousal: Wie hoch ist das Erregungsniveau des Patienten?

- schläfrig, Koma ähnlich (1)
- ruhig (2)
- neutral (3)
- aufgeregt (4)
- übererregt (5)

Q29 Die Richtung: Zeigt der Patient eher Annäherung oder Vermeidung an andere Personen oder Objekte?

- abwehrend (1)
- sich entfernend (2)
- neutral (3)
- sich annähernd (4)
- sich anklammernd (5)

Q30 Zeigt der Patient einen Gefühlsausbruch

- Weinen (1)
- Lachen (2)
- Aggression (3)
- Zuneigung (4)
- anders, definiere: (5) ________________________________________________
- nein (6)

*Display This Question: If Sind neben dem Patienten noch andere Personen anwesend? (hier zählt auch die filmende Person) != Nein*

Q31 Erzeugen die anderen Personen Geräusche oder sprechen sie?

- Ja (1)
- Nein (2)

*Display This Question: If Erzeugen die anderen Personen Geräusche oder sprechen sie? = Ja*

Q32 Entnimmst du den Geräuschen emotionale Bedeutung?

- Ja (1)
- Nein (2)

*Display This Question: If Erzeugen die anderen Personen Geräusche oder sprechen sie? = Ja*

Q33 Drückt die Person sich in Worten aus?

- Ja (1)
- Nein (2)

*Display This Question: If Drückt die Person sich in Worten aus? = Ja*

Q34 Was ist der Inhalt?

- Krankheit, Anfall (1)
- Vorgang des Filmens (2)
- anders, definiere: (3) ________________________________________________

*Display This Question: If Drückt der Patient sich in Worten aus? = Ja*

Q35 Was ist der Inhalt?

- Empfindungen während des Anfalls (1)
- Vorgang des Filmens (2)
- anders, definiere: (3) ________________________________________________

Q36 Spricht die Person den Patienten direkt an?

- Ja (1)
- Nein, spricht über Patienten (2)

*Display This Question: If Spricht die Person den Patienten direkt an? = Ja*

Q37 Antwortet der Patient oder führt eine geforderte Aktion aus?

- Ja (1)
- Nein (2)

Q46 Schätze das emotionale Erleben der anderen Person(en) ein:
Die Valenz: Ist die Wertigkeit der Emotion positiv oder negativ?

- sehr unangenehm (1)
- eher unangenehm (2)
- Neutral (3)
- eher angenehm (4)
- sehr angenehm (5)

Q47 Das Arousal: Wie hoch ist das Erregungsniveau der anderen Personen?

- schläfrig (1)
- ruhig (2)
- neutral (3)
- aufgeregt (4)
- übererregt (5)

Q48 Die Richtung: Zeigen die anderen Personen eher Annäherung oder Vermeidung an Andere oder Objekte?

- Abwehr (1)
- sich entfernen (2)
- neutral (3)
- sich annähern (4)
- sich anklammern (5)

Q49 Zeigen die Personen einen Gefühlsausbruch?

- Weinen (1)
- Lachen (2)
- Aggression (3)
- Zuneigung (4)
- anders, definiere: (5) ________________________________________________
- nein (6)

*Display This Question: If Sind neben dem Patienten noch andere Personen anwesend? (hier zählt auch die filmende Person) != Nein*

Q54 Wie ist das interaktive Verhalten zwischen Patient und anderen Personen?

- nonverbal (1)
- nonverbal & verbal (2)
- keins (3)

*Display This Question: If Wie ist das interaktive Verhalten zwischen Patient und anderen Personen? != keins*

Q55 Gibt es Augenkontakt zwischen dem Patient und anderen Personen oder der Kamera?

- Ja (1)
- Nein (2)

*Display This Question: If Sind neben dem Patienten noch andere Personen anwesend? (hier zählt auch die filmende Person) != Nein*

Q56 Gibt es Körperkontakt zwischen dem Patient und einer anderen Person?

- Ja (1)
- Nein (2)

*Display This Question: If Sind neben dem Patienten noch andere Personen anwesend? (hier zählt auch die filmende Person) != Nein*

Q57 Kümmert sich jemand um den Patienten?

- Ja (1)
- Nein (2)

*Display This Question: If Wie ist das interaktive Verhalten zwischen Patient und anderen Personen? != keins*

Q58 Hat die Interaktion einen Einfluss auf den Anfall?

- Ja (1)
- Nein (2)
- Kann ich nicht sagen (3)

*Display This Question: If Hat die Interaktion einen Einfluss auf den Anfall? = Ja*

Q59 Welchen Einfluss hat die Interaktion auf den Anfall?

- verstärkend (1)
- vermindernd (2)
- kann ich nicht sagen (3)

Q66 Kannst du verstärkende Aspekte ("Belohnungen") oder vermindernde Aspekte ("Bestrafungen") ausmachen?

- Ja, definiere: (1) ________________________________________________
- Nein (2)

Q60 Möchtest du noch etwas zu der Interaktion zwischen dem Patient und Anderen hinzufügen?

________________________________________________________________

Q61 Wie aufmerksam warst du beim Betrachten des Videos?

- Meine Aufmerksamkeit war auf das Video fokussiert. (1)
- Es fiel mir schwer meine Aufmerksamkeit bei dem Video zu halten. (2)
- Ich war abgelenkt. (3)

Q62 Wirkt der Patient hilfsbedürftig und/oder erweckt er Mitleid?

- Ja (1)
- Nein (2)

Q63
Bitte schätze im Folgenden dein eigenes emotionales Erleben ein:
Die Valenz: Ist die Wertigkeit deiner Emotion positiv oder negativ?

- sehr unangenehm (1)
- eher unangenehm (2)
- Neutral (3)
- eher angenehm (4)
- sehr angenehm (5)

Q64 Das Arousal: Wie hoch ist dein Erregungsniveau beim Betrachten der Videos?

- schläfrig (1)
- ruhig (2)
- neutral (3)
- aufgeregt (4)
- übererregt (5)

Q65 Die Richtung: Erzeugt deine Emotion Annäherung oder Vermeidung?

- Abwehr (1)
- sich entfernen (2)
- neutral (3)
- sich annähern (4)
- sich anklammern (5)

Q66 Hattest du einen Gefühlsausbruch?

- Weinen (1)
- Lachen (2)
- Aggression (3)
- Zuneigung (4)
- anders, definiere: (5) ________________________________________________
- nein (6)

67 Ist auch das Verhalten nach dem Anfall sichtbar?

- Ja (1)
- Nein (2)

*Display This Question: If Ist auch das Verhalten nach dem Anfall sichtbar? = Ja*

Q68 Zeigt der Patient Anzeichen von Verwirrtheit NACH dem Anfall?

- Ja (1)
- Nein (2)

*Display This Question: If Ist auch das Verhalten nach dem Anfall sichtbar? = Ja*

Q67 Erinnert der Patient sich an den Anfall?

- Ja (1)
- Nein (2)
- Kann ich nicht sagen (3)

*Display This Question: If Ist auch das Verhalten nach dem Anfall sichtbar? = Ja*

Q69 Zeigt der Patient Anzeichen von Verhaltensproblemen oder Ruhelosigkeit NACH dem Anfall?

- Ja (1)
- Nein (2)

*Display This Question: If Ist auch das Verhalten nach dem Anfall sichtbar? = Ja*

Q70 Erholt sich der Patient zügig von dem Anfall?

- Ja (1)
- Nein (2)

*Display This Question: If Ist auch das Verhalten nach dem Anfall sichtbar? = Ja*

Q68 Wie ist die Atemfrequenz des Patienten nach dem Anfall?

- tief (1)
- oberflächlich (2)

*Display This Question: If Ist auch das Verhalten nach dem Anfall sichtbar? = Ja*

Q69 Wie ist die Lautstärke der Atmung des Patienten nach dem Anfall?

- laut/schnarchend (1)
- leise (2)

*Display This Question: If Ist auch das Verhalten nach dem Anfall sichtbar? = Ja*

Q70 Wie ist regelmäßig ist die Atmung des Patienten nach dem Anfall?

- regelmäßig (1)
- unregelmäßig (2)

*Display This Question: If Ist auch das Verhalten nach dem Anfall sichtbar? = Ja*

Q61 Beschreibe die Situation nach dem Anfall:

________________________________________________________________

Q65
Fallen dir nun noch andere Bilder, Vergleiche oder Beschreibungen ein, um die Situation darzustellen?
Hier hast du dir Möglichkeit deine Beschreibung vom Anfang zu ergänzen.

**APPENDIX B**

**Table B1**

Coefficients of the backward model predicting diagnosis (ES/FDS).

| Included | b | 95% CI for Odds Ratio | | |
| --- | --- | --- | --- | --- |
|  |  | Lower | Odds | Upper |
| Constant | -1.37 |  |  |  |
| Eyes closed | 1.47*** | 2.57 | 4.36 | 7.40 |
| Eye-contact between patient and others/camera | -0.98*** | 0.22 | 0.37 | 0.64 |
| Patient awake | 0.99 | 0.95 | 2.70 | 7.70 |
| Repetitive movement | 0.48 | 0.98 | 1.62 | 2.68 |
| Music in background | 1.47** | 1.56 | 4.34 | 12.07 |
| Private environment | -0.81 | 0.17 | 0.45 | 1.14 |

Note. R² = .15 (Cox&Snell), .23 (Nagelkerke). Model Χ²(6) = 66.51, p < .001. * < .05, ** < .01, *** < .001

**Table B2**

Classification table.

| Observed diagnosis | Predicted diagnosis | | |
| --- | --- | --- | --- |
|  | ES | FDS | % correct |
| ES | 283 | 16 | 94,6% |
| FDS | 68 | 32 | 32,0% |
|  |  |  | 78,9% |
