# Supplementary material for: Functionality of symptoms and interpersonal communication in home video recordings of functional/dissociative versus epileptic seizures
Source: Epilepsia. 2026 Feb 5;67(5):2241–53. doi: 10.1002/epi.70107 (PMC13179661; doi:10.1002/epi.70107)
Supplement: Supplementary file 1 — Appendix S1. [file EPI-67-2241-s001.zip › epi70107-sup-0002-Table3-Suppl-Inform-new.docx]

**Table 3**

Inter-Rater Reliabilities

| 1. **Context variables** | **overall IRR** | **IRR ES only** | **IRR FDS only** |
| --- | --- | --- | --- |
| - 1. Patient in recumbent position | *κ* = .74** | *κ* = .73** | *κ* = .73** |
| - 1. Patient lying | *κ* = .78** | *κ* = .80** | *κ* = .70** |
| - 1. Private or public setting | *κ* = .86** | *κ* = .86** | *κ* = .85** |
| - 1. Background TV | *κ* = .69** | *κ* = .74** | *κ* = .57* |
| - 1. Background music | *κ* = .49* | *κ* = .48* | *κ* = .48* |
| - 1. Background conversation | *κ* = .42* | *κ* = .39 | *κ* = .47* |
| - 1. Day or night | *κ* = .48* | *κ* = .47* | *κ* = .50* |
| - 1. Other persons present | *κ* = .75** | *κ* = .72** | *κ* = .81** |
| - 1. Pets present | *κ* = .70** | *κ* = .72** | *κ* = .67** |
| - 1. . Contact between patient and pet (if visible) | *κ* = .41* | *κ* = .43* | *κ* = .21 |
| 1. **Patient variables** | **overall IRR** | **IRR ES only** | **IRR FDS only** |
| 2.1. Patient flees | *κ* = .14 | *κ* = .16 | *κ* = .05 |
| 2.2. Patient is aggressive | *κ* = .14 | *κ* = .16 | *κ* = .05 |
| 2.3. Patient is ready to fight (alert), not moving | *κ* = .14 | *κ* = .16 | *κ* = .05 |
| 2.4. Patient is dissociated, not moving | *κ* = .24 | *κ* = .13 | *κ* = .36 |
| 2.5. Patient moves | *κ* = .26 | *κ* = .20 | *κ* = .35 |
| 2.6. Face is moving (if moving yes) | *κ* = .21 | *κ* = .15 | *κ* = .23 |
| 2.7. Head is moving (if moving yes) | *κ* = .25 | *κ* = .23 | *κ* = .26 |
| 2.8. Trunk is moving (if moving yes) | *κ* = .30 | *κ* = .26 | *κ* = .39 |
| 2.9. Arms moving (if moving yes) | *κ* = .35 | *κ* = .31 | *κ* = .41* |
| 2.10. Legs moving (if moving yes) | *κ* = .58* | *κ* = .57* | *κ* = .62** |
| 2.11. Movement restricted to one side (if moving yes) | *κ* = .08 | *κ* = -.02 | *κ* = .00 |
| 2.12. Repetitive movement (if moving yes) | *κ* = .46* | *κ* = .49* | *κ* = .31 |
| 2.13. Synchrony of movement (if moving yes) | *κ* = .40 | *κ* = .36 | *κ* = .39 |
| 2.14. Increasing intensity (if moving yes) | *κ* = .03 | *κ* = .01 | *κ* = .07 |
| 2.15. Decreasing intensity (if moving yes) | *κ* = .10 | *κ* = .09 | *κ* = .13 |
| 2.16. Dynamic movement (if moving yes) | *κ* = .12 | *κ* = .05 | *κ* = .20 |
| 2.17. No change in intensity (if moving yes) | *κ* = .22 | *κ* = .19 | *κ* = .23 |
| 2.18. Movement from side-to-side (if moving yes) | *κ* = .29 | *κ* = .18 | *κ* = .45* |
| 2.19. Eyes closed | *κ* = .61** | *κ* = .52* | *κ* = .65** |
| 2.20. Apparent injury | *κ* = .16 | *κ* = .20 | *κ* = -.00 |
| 2.21. Visible body reactions (blushing, sweating,…) | *κ* = .24 | *κ* = .23 | *κ* = .23 |
| 2.22. Patient seems awake | *κ* = .50* | *κ* = .53* | *κ* = .53* |
| 2.23. Patient is producing sounds | *κ* = .59* | *κ* = .56* | *κ* = .66** |
| 2.24. Sounds have emotional meaning (if sound yes) | *κ* = .39 | *κ* = .41* | *κ* = .28 |
| 2.25. Patient uses words (if sound yes) | *κ* = .68** | *κ* = .72** | *κ* = .55* |
| 2.26. Content of vocalization: seizure (if sound yes) | *κ* = .48* | *κ* = .46* | *κ* = .52* |
| 2.27. Content of vocalization: filming (if sound yes) | *κ* = .49* | *κ* = .44* | *κ* = .56* |
| 2.28. Emotional outburst | *κ* = .53* | *κ* = .49* | *κ* = .60* |
| 2.29. Emotional valence | α = .74* | α = .72* | α = .76** |
| 2.30. Emotional arousal | α = .69* | α = .69* | α = .68* |
| 2.31. Emotional direction | α = .68* | α = .74* | α = .39* |
| 1. **Variables relating to other persons** | **overall IRR** | **IRR ES only** | **IRR FDS only** |
| 3.1. Others producing sounds | *κ* = .72** | *κ* = .70** | *κ* = .75** |
| 3.2. Sounds have emotional meaning (if sound yes) | *κ* = .29 | *κ* = .35 | *κ* = .14 |
| 3.3. Others use words (if sound yes) | *κ* = .23 | *κ* = .28 | *κ* = -.01 |
| 3.4. Content of vocalization: seizure (if sound yes) | *κ* = .54* | *κ* = .53* | *κ* = .54* |
| 3.5. Content of vocalization: filming (if sound yes) | *κ* = .39 | *κ* = .34 | *κ* = .50* |
| 3.6. Emotional outburst | *κ* = .26 | *κ* = .27 | *κ* = .24 |
| 3.7. Emotional valence | α = .51* | α = .55* | α = .47 |
| 3.8. Emotional arousal | α = .52* | α = .51* | α = .55* |
| 3.9. Emotional direction | α = .67* | α = .65* | α = .69* |
| 1. **Interactional variables**   **(only if other person present)** | **overall IRR** | **IRR ES only** | **IRR FDS only** |
| 4.1. Other talking to the patient | *κ* = .62** | *κ* = .58* | *κ* = .71** |
| 4.2. Other talking about patient | *κ* = .53* | *κ* = .53* | *κ* = .53* |
| 4.3. Patient answering or reacting | *κ* = .59* | *κ* = .54* | *κ* = .69** |
| 4.4. Only nonverbal interaction | *κ* = .22 | *κ* = .26 | *κ* = .13 |
| 4.5. Nonverbal and verbal interaction | *κ* = .47* | *κ* = .47* | *κ* = .47* |
| 4.6. Eye contact | *κ* = .43* | *κ* = .43 | *κ* = .43* |
| 4.7. Body contact | *κ* = .64** | *κ* = .63** | *κ* = .67** |
| 4.8. Caring behavior | *κ* = .54* | *κ* = .48* | *κ* = .68** |
| 4.9. Interaction influences seizures | *κ* = .09 | *κ* = .14 | *κ* = -.02 |
| 4.10. Interaction reinforces seizure | *κ* = .06 | *κ* = .05 | *κ* = .09 |
| 4.11. Interaction decreases seizure | *κ* = .12 | *κ* = .16 | *κ* = .07 |
| 4.12. Patient is in need of help | *κ* = .24 | *κ* = .28 | *κ* = .15 |
| 1. **Rater-related variables** | **overall IRR** | **IRR ES only** | **IRR FDS only** |
| 5.1. Emotional outburst | *κ* = .02 | *κ* = .02 | *κ* = .01 |
| 5.2. Emotional valence | α = .47* | α = .46* | α = .48* |
| 5.3. Emotional arousal | α = .26 | α = .12 | α = .47* |
| 5.4. Emotional direction | α = .26 | α = .22 | α = .36 |

Note: * = moderate inter-rater reliability; ** = high inter-rater reliability; Note the different classification of IRR values for Cohen’s kappa and Cronbach’s alpha as described in the methods section.
